# Zinc Deficiency Leads to Lipid Changes in *Drosophila* Brain Similar to Cognitive‐Impairing Drugs: An Imaging Mass Spectrometry Study

**DOI:** 10.1002/cbic.202000197

**Published:** 2020-06-16

**Authors:** Mai H. Philipsen, Chaoyi Gu, Andrew G. Ewing

**Affiliations:** ^1^ Department of Chemistry and Molecular Biology University of Gothenburg Kemigården 4 412 96 Göteborg Sweden; ^2^ Department of Chemistry and Chemical Engineering Chalmers University of Technology Kemigården 4 412 96 Göteborg Sweden

**Keywords:** cognition, *Drosophila melanogaster*, lipid changes, mass spectrometry imaging, zinc deficiency

## Abstract

Several diseases and disorders have been suggested to be associated with zinc deficiency, especially learning and memory impairment. To have better understanding about the connection between lipid changes and cognitive impairments, we investigated the effects of a zinc‐chelated diet on certain brain lipids of *Drosophila melanogaster* by using time‐of‐flight secondary ion mass spectrometry (ToF‐SIMS). The data revealed that there are increases in the levels of phosphatidylcholine and phosphatidylinositol in the central brains of the zinc‐deficient flies compared to the control flies. In contrast, the abundance of phosphatidylethanolamine in the brains of the zinc‐deficient flies is lower. These data are consistent with that of cognitive‐diminishing drugs, thus providing insight into the biological and molecular effects of zinc deficiency on the major brain lipids and opening a new treatment target for cognitive deficit in zinc deficiency.

## Graphical abstract

**No zinc, no think**: Using mass spectrometric imaging, we examined how dietary zinc chelation can alter lipid composition and lipid abundance in the central brain of *Drosophila*. The increased PC and PI levels and the decreased PE level induced by zinc deficiency resemble the effects of several cognitive‐impairing drugs, including cocaine and ketamine.

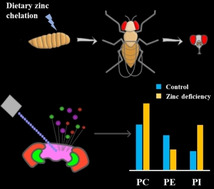

Zinc is an essential trace element that plays important roles in various functions of the central nervous system, such as modulation of neurotransmitter activity, enzymatic activity, and cellular signaling.^1^ Zinc is highly abundant in the brain and a high fraction of zinc ions either bind to proteins or are required for enzymatic functions. As zinc has been shown to bind approximately 10 % of human proteins, a lack of this trace element leads to severe problems in brain functions during growth and development.^2^ Zinc deficiency has been suggested to induce oxidative stress as well as disrupt growth factor signaling molecules, and thus induce neuronal apoptosis.^3^ In addition to that, zinc deficiency during pregnancy leads to elevated risk of abortion, low birth weight and negative effects on the birth process.^4^ Zinc accumulates significantly in the hippocampus in brain and is essential for hippocampus‐dependent memory.^5^ Although chronic zinc deficiency affects signaling molecules that are involved in memory formation, other possible pathways through which zinc deficiency impairs learning and memory remain to be understood.^6^ Lipids, the major component of cellular membranes, are shown to be altered by cognition‐related drugs.^7^ Additionally, the effects of zinc deficiency on lipid compositions have been demonstrated in rats and cells.^8^ Therefore, we have examined the possible correlation between lipid alterations and zinc deficiency‐induced memory impairment.

Many analytical methods are available for analysis of lipidomics, including mass spectrometric imaging (MSI), liquid chromatography−mass spectrometry, and other mass spectrometry‐based techniques.7a, 9 Among them, MSI is a powerful tool to probe and provide spatial information of lipid species within biological samples. ToF‐SIMS, a label‐free MSI technique, has been used in a range of biomedical studies on cells and tissues due to its low detection limit and high spatial resolution. Recently, ToF‐SIMS has been applied as an approach to analyze lipidomics in diseases and disorders, including cancer, fatty liver, cardiovascular disease, Alzheimer's disease, and so on.^10^ In addition to that, certain medications or diets have also been shown to be able to induce changes in lipid structures and composition in the brain.9d, 11 In this study, we investigated the alterations of lipids in the fly brain caused by dietary zinc chelation using ToF‐SIMS. ToF‐SIMS imaging was performed using the ToF SIMS V (ION‐TOF GmbH) with a 25 keV Bi_3_^++^ primary ion source. The use of a cluster primary ion beam provides the possibility to obtain images of tissue sections at micron spatial resolution and spectra with a wide mass range, while the sample preparation remains relatively simple.

*Drosophila melanogaster*, the fruit fly, is an attractive model for studying zinc deficiency as the machinery for transporting and regulating zinc in *Drosophila* is similar to that in mammals.^12^ Moreover, the metal‐responsive transcription factor‐1 and its transcriptional targets, which are important in maintaining cellular zinc homeostasis in mammals to avoid heavy‐metal‐induced toxicity, are discovered in flies.^13^

Zinc deficiency in flies can be induced by two methods, genetic manipulation or modification of standard fly food with chelating agents that show high affinity for zinc, such as *N,N,N′*,*N′*‐tetrakis(2‐pyridinylmethyl)ethanediamine (TPEN).^14^ Although TPEN also has effects on other metal ions, including iron and copper, the iron level in flies are not significantly affected by food supplemented with TPEN.14c, 15 In our experiments, 100 μM TPEN was used to chelate a portion of zinc from the standard fly food to generate zinc‐chelated food, whereas control flies were given the standard food throughout the entire life cycle. Even though it was clearly observed that the growth of the flies fed with zinc‐chelated food was retarded, how much zinc remained inside the fly was unclear. To answer this question, inductively coupled plasma mass spectrometry (ICP‐MS) was performed to quantify the concentration of zinc in control as well as zinc‐deficient larvae and flies (Figure S1 in the Supporting Information). We found that the average concentration of zinc in control and zinc‐deficient larvae were 941.01 and 312.29 ppb, respectively, meaning that the zinc‐chelated food effectively reduced <B2/3> of the total zinc level inside the fly larvae. Moreover, zinc‐chelated food also cut the total zinc level in the body of the adult fly to half. This decreased level of zinc might be due to that the chelated zinc is more readily excreted by the flies. It is noticeable that the concentration of zinc inside the head of the adult fly is much lower than the concentration in the fly body. However, by feeding the flies with zinc‐chelated food, the zinc concentration in the fly head decreased but not significantly (decreased from 284.57 to 214.96 ppb). In *Drosophila*, dietary zinc is stored in Malpighian tubules and the expression of *Zip71B*, the protein responsible for zinc detoxification and importing zinc across the membrane, can be regulated with dietary zinc to maintain zinc homeostasis inside the fly body.^16^ The total zinc level in the brain, on the other hand, has been suggested to be more tightly controlled and barely affected by peripheral zinc level.^17^ This might explain why zinc‐chelated diet influences zinc concentration in the fly body more significantly than in the fly brain.

Prior to ToF‐SIMS analysis, flies were placed on fly collars and frozen in order to separate the heads from the bodies. The heads were subsequently sectioned into thin slices with a thickness of 12 μm under −20 °C and thaw‐mounted on glasses coated with indium tin oxide (ITO). The sliced samples were then freeze‐dried under vacuum right before the analysis and ToF‐SIMS equipped with a 25 keV Bi_3_^++^ primary ion beam was used for the entire analysis. Figure 1 shows the ion images of phosphatidylcholine (PC) fragments at *m*/*z* 184.1 and 224.1 as well as phosphatidylethanolamine (PE) fragment at *m*/*z* 140.0 obtained from ToF‐SIMS analysis. PC and PE fragments are caused by the bombardment of primary ion beam on the surface of the sample. Zinc deficiency leads to increases of the intensities of the PC fragments at *m*/*z* 184.1 and 224.1 in the entire fly head, particularly in the area of the central brain, whereas the zinc chelated diet induces a decrease of the intensity of PE fragment at *m*/*z* 140.0, and this decrease is more obvious in the central brain region and the optical lobula.

**Figure 1:**
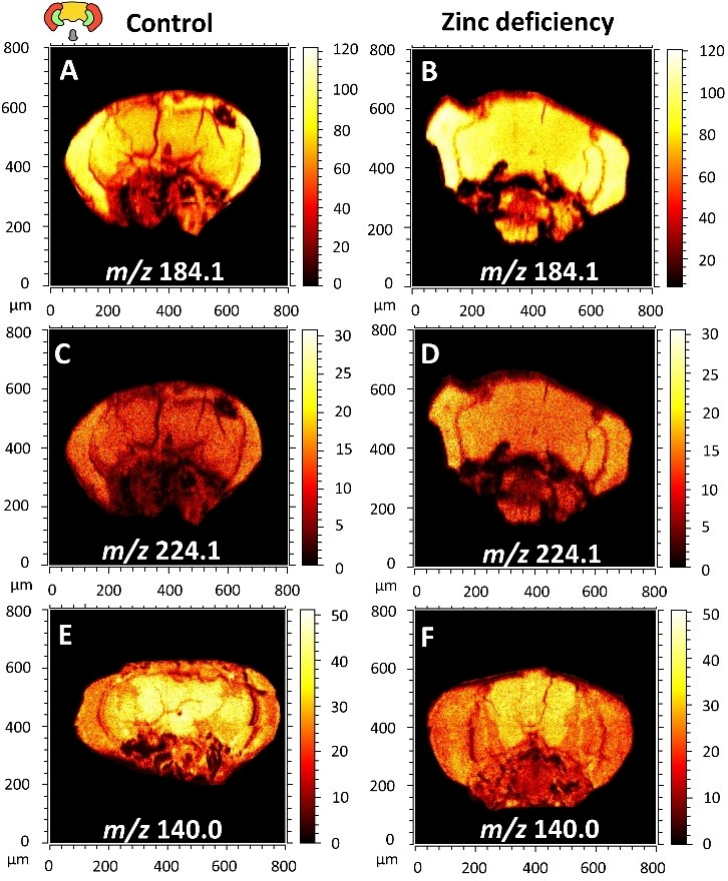
Distribution of PC fragments at *m*/*z* 184.1 and 224.1 in positive‐ion mode and PE fragment at *m*/*z* 140.0 in negative‐ion mode in the brains of control and zinc‐deficiency flies. All data were acquired by using ToF‐SIMS equipped with a 25 keV Bi_3_^++^, and the total ion dose was 1.5 x 10^12^ ions/cm^2^. The symbolic image on the top left illustrates the structure of the fly head, including medulla of optic lobes (the two red parts), optical lobula (the two green parts), the central brain (the yellow region in the middle) and proboscis (the gray area).

To obtain specific information regarding how dietary zinc chelation affects the brain, we imaged the area of the central brains of fly heads to perform data analysis. As the spectra from ToF‐SIMS experiments contain much higher intensities of lipid fragments than lipid molecules, specifically in the mass region with *m*/*z* less than 300, principal component analysis (PCA) was performed only in the mass range between 650 and 900 to examine molecular ions. PCA is a multivariate data analysis method and can be applied to analyze variations within the lipid region between samples of control and zinc‐deficient flies. The image output score in positive ion mode is plotted in Figure 2A and reveals the separation between control and zinc‐deficient groups along principal component 3. The corresponding loading plot, which is shown in Figure 2B, demonstrates the peaks contributing to the separation of these two groups. Elevated intensities of various PC species and their salt adducts, including PC (32 : 1) at *m*/*z* 732.6, PC (34 : 2) at *m*/*z* 758.6, PC (34 : 1) at *m*/*z* 760.6, PC (36 : 4) at *m*/*z* 782.6, PC (34 : 1)+K at *m*/*z* 798.5 and PC (36 : 2)+Na at *m*/*z* 808.6, are observed after zinc chelated diet. These observations are consistent with the increased intensity of PC fragments at *m*/*z* 184.1 in the samples of fly head upon zinc deficiency. In contrast, the levels of triacylglycerols (TAGs) are more dominant in control samples, such as TAG (48:0) at *m*/*z* 807.6 and TAG (46 : 1)+K at *m*/*z* 815.7.

**Figure 2:**
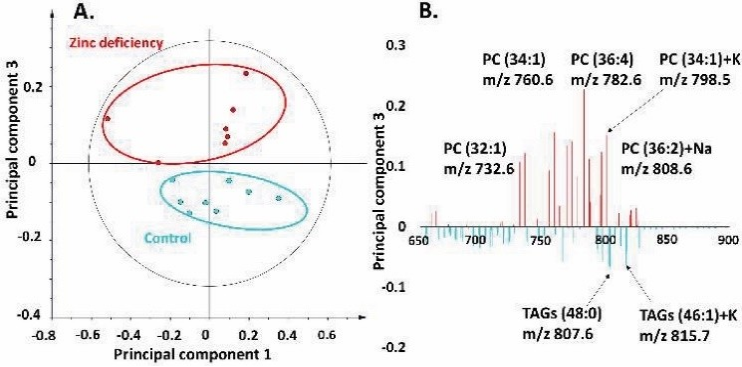
PCA of positive‐ion‐mode data of the central brain regions of control versus zinc‐deficient flies from the ToF‐SIMS experiment. A) Score plot of principal component 1 versus principal component 3 from the spectra. B) Corresponding loading plot of principal component 3 showing the peaks that contribute to the separation of the two fly groups.

In negative‐ion mode, the control and the zinc‐deficient groups are again clearly separated along principal component 3, as can be seen in Figure 3A. The associated loading plot of principal component 3, as plotted in Figure 3B, shows the PE and phosphatidylinositol (PI) species that are responsible for the separation in this principal component. PI species, such as PI (32 : 1) at *m*/*z* 807.5 and PI (34 : 1) at *m*/*z* 835.5, are less abundant in control flies. Similar to PI species, the reductions of PE (34 : 1) at *m*/*z* 716.5 and PE (34 : 2) at *m*/*z* 714.5 are observed in the central brain of the control group as well. In contrast, the control flies have higher level of PE (36 : 1) at *m*/*z* 744.5 as well as PE (36 : 2) at *m*/*z* 742.5 compared to the zinc‐deficient flies.

**Figure 3:**
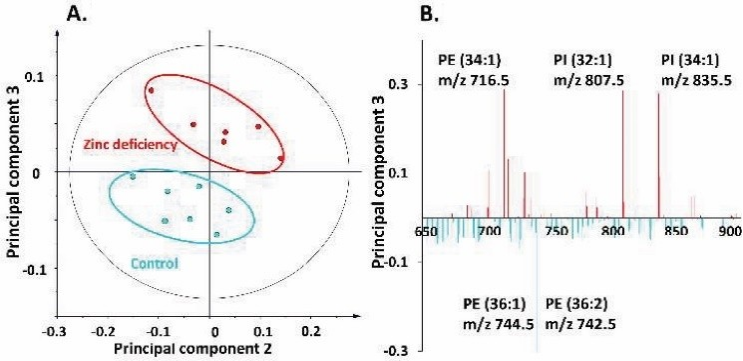
PCA of negative‐ion‐mode data of the central brain regions of control versus zinc‐deficient flies from the ToF‐SIMS experiment. A) Score plot of principal component 2 versus principal component 3 from the spectra. B) Corresponding loading plot of principal component 3 showing the peaks that contribute to the separation of the two fly groups.

Based on the results obtained from PCA, the relative intensities of the lipid species in central fly brain which contribute to the separation in the PCA were examined and these are illustrated in Figure 4. Unsaturated PC species, which are detected as [*M*+H]^+^, and their salt adducts have increased intensities upon dietary zinc chelation. In negative‐ion mode, the increases of the abundance of a variety of PI species in the central brain region, such as PI (32 : 1) at *m*/*z* 807.5, PI (32 : 0) at *m*/*z* 809.6, PI (34 : 2) at *m*/*z* 833.5, PI (34 : 1) at *m*/*z* 835.5 and PI (34 : 0) at *m*/*z* 837.6, are induced by the diet with zinc chelation. Moreover, the signal of the PI fragment at *m*/*z* 241.0, which is related to the lipid species of PI, is also elevated in the central brains of the flies fed with the zinc chelated diet. For PE lipid species, the data in Figure 4 show that the abundance of PE with 34 carbons in the fatty acid tails increases, whereas the zinc chelated diet lowers the intensities of PE with 36 carbons in the fatty acid tails.

**Figure 4:**
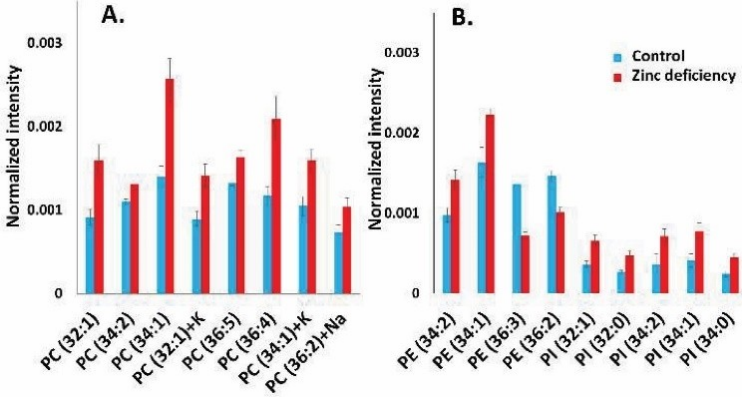
Relative quantification of A) PC species and B) PE and PI species in the central brains of control and zinc‐deficient flies. Images were obtained in positive‐ and negative‐ion modes by using 25 keV Bi_3_^++^ as the primary ion source. Data were collected from 24 fly heads for both groups with each measurement repeated 3 times. Peak intensities were normalized to numbers of selected pixels and total peak intensity. The error bars represent SEM and show variations among different fly heads. PC species were detected as [*M*+H]^+^ unless specified as Na/K adduct species. PE and PI species were detected as [*M*−H]^−^.

Dietary zinc chelation induces the elevations of PC and PI levels and the depletion of total PE level in the central fly brain. Consistent with our data, Carman et al. reported an increased level of PI and decreased level of PE in cells upon depletion of zinc.8b The activities of most enzymes in the CDP‐diacylglycerol pathway, including phosphatidylserine synthase and decarboxylase, appear to be decreased in response to zinc deficiency and thus, this condition results in a decreased PE level.^18^ In contrast, by upregulating the activity of PI synthase, zinc deficiency elevates the level of PI. Moreover, the abundance of PC is significantly increased upon zinc deficiency because of the enhanced activity of the choline phosphotransferase.^19^

Previous studies on neuroendocrine cells reveal that zinc treatment increases the levels of PC lipid species while it decreases the abundance of PEs in the cellular membrane, which resembles the changes of lipid species caused by zinc deficiency shown here for *Drosophila* brain.^20^ The changes observed for PIs, however, appear to be opposite to these between zinc treatment and zinc deficiency. The TPEN post‐treatment brought the levels of the lipids slightly but not completely back to the control level, demonstrating apparently irreversible effects to the cellular membrane induced by zinc treatment. It is well accepted that zinc deficiency can cause the impairment of learning and memory, but the mechanisms regarding how zinc administration influences cognitive process are not fully understood yet. It seems that zinc administration might have biphasic effects, with high concentration being neurotoxic and low concentration being neuroprotective, especially for post injury conditions.^21^

In agreement with our data, cognitive impairing drugs, such as ketamine and cocaine, induce similar changes in lipid composition compared to zinc deficiency. Acute and chronic usage of ketamine, a medication that is used as an anesthetic as well as a treatment for depressive disorder, has been shown to exhibit negative effects on cognition and by treating the depressive disorder patients with ketamine, increased concentrations of phospholipid, particularly PC, were observed.^22^ The effects of zinc deficiency on the abundance of certain lipid species are similar to the effects induced by the administration of cocaine, a drug that leads to a decline in cognition.7a To be more specific, these similar effects include that both zinc deficiency and cocaine administration elevate the levels of PC species as well as PE (34 : 1), PE (34 : 2), PI (34 : 1) and PI (34 : 2), while they both reduce the signals of several other PE species, including PE (36 : 3) and PE (36 : 2). In contrast, methylphenidate, a psychostimulant that has similar action as cocaine on the dopamine transporter but appears to enhance cognitive performance and memory, changes the intensities of lipid species opposite to cocaine.7a

In mammals, zinc is stored in synaptic vesicles and is released together with glutamate during exocytosis of glutamatergic neurons. This released zinc can subsequently modulate certain receptors and ion channels, suggesting the importance of zinc in regulating learning and memory.1b, 23 Zinc deficiency caused by diet is linked to cognitive impairment and different pathways underlying this have been proposed.^24^ Based on our study, it seems quite possible that in the brain, alterations of the abundance of certain lipid species are related to improvement or impairment in cognition. Our study, when compared to other studies focusing on cognition‐related drugs, suggests that the cognitive loss in the brain induced by zinc deficiency could lead to an increased amount of PC, 34‐carbon chain PE, and 34‐carbon chain PI, as well as a decreased amount of 36‐carbon chain PE.

## Conflict of interest

The authors declare no conflict of interest.

## Supporting information

As a service to our authors and readers, this journal provides supporting information supplied by the authors. Such materials are peer reviewed and may be re‐organized for online delivery, but are not copy‐edited or typeset. Technical support issues arising from supporting information (other than missing files) should be addressed to the authors.

Supplementary
