# Supplementary material for: Zinc Deficiency Leads to Lipid Changes in Drosophila Brain Similar to Cognitive‐Impairing Drugs: An Imaging Mass Spectrometry Study
Source: Chembiochem. 2020 Jun 16;21(19):2755–8. doi: 10.1002/cbic.202000197 (PMC7586942; doi:10.1002/cbic.202000197)
Supplement: Supplementary file 1 — Supplementary [file CBIC-21-2755-s001.pdf]

ChemBioChem

Supporting Information

# Zinc Deficiency Leads to Lipid Changes in *Drosophila* Brain Similar to Cognitive-Impairing Drugs: An Imaging Mass Spectrometry Study

Mai H. Philipsen[+], Chaoyi Gu[+], and Andrew G. Ewing*

## Table of contents

Experimental section ........ 3

Figure S1 ........ 4

Figure S2 ........ 5

## Experimental Section

**Materials**

Chemicals including TPEN and gelatin were purchased from Sigma-Aldrich (Sweden). Deionized water was collected from a Milli-Q water system (Millipore, Merck, Darmstadt, Germany).

**Fly strains and zinc deficient food**

The wild-type Canton S strain flies were used for the entire experiments. All the files were maintained at room temperature (23-26 ºC) with a 12 h light/dark cycle. 15 pairs of two- to three-day-old male and female flies were picked for each control and zinc deficient group. The control groups were fed with standard fly food and the zinc deficient groups were fed with standard fly food supplemented with 100 µM TPEN to chelate zinc. The flies were allowed to lay eggs on the food for 5 days and the male offspring were used for the sample preparation.

**ICP-MS sample preparation and analysis**

The male adult flies from both groups were snap frozen in liquid nitrogen and vortexed to separate the heads from the bodies. The adult heads, adult bodies and larvae were digested separately with 1 mL 65% nitric acid (Supra pure quality, Merck, Darmstadt, Germany) overnight. The 0.1 M nitric acid solution was prepared by diluting 65% nitric acid with Milli-Q water. Prior to the ICP-MS analysis, the solutions of digested larvae and adult bodies for the control group were diluted 80 times with Milli-Q water. The solutions of digested adult heads for the control group, as well as the larvae, adult heads and adult bodies for the zinc deficient group were diluted 40 times. All solutions were then filtered using 0.45 μm filters (VWR, Sweden). All materials, including vessels, pipets, and plastic tubes, were rinsed with Milli-Q water to avoid contaminants. Zinc standard, which was obtained as 10000 ppb aqueous solution from CPA Chem standard, was diluted with 0.1 M nitric acid to final concentrations of 0, 1, 10 and 50 ppb to construct calibration curve. Yttrium was used as an internal standard. ICP-MS analysis was performed using a model iCAP-Q ICP-MS (Thermo Fisher, Cambridge, UK).

**Sample preparation for ToF-SIMS imaging**

The male adult flies from both groups were placed on the same fly collars (4 M Instrument and Tool LLC) with all the heads facing the same direction. The fly collars were then embedded in 10% gelatin solution and frozen in a -80 °C freezer for 1 h. The frozen heads were subsequently removed from the fly collars and stored in liquid nitrogen. Prior to the ToF-SIMS analysis, the frozen fly heads were sectioned into 12-µm-thickness slices at -20 °C using a microtome (Cryostat Leica CM 1520). The slices were then thaw-mounted on ITO-coated glass slides and freeze-dried inside the ToF-SIMS instrument overnight.

**ToF-SIMS imaging**

ToF-SIMS analysis was performed using TOF.SIMS V (ION-TOF GmbH) equipped with bismuth (Bi) liquid metal ion gun as the primary ion source. The images were acquired in both positive and negative ion modes using the 25 keV $Bi_3^{++}$ primary ions over an area of 500 x 500 µm$^2$ with 512 x 512 pixels. A mass resolution of $\Delta m/m$ 6000 at *m/z* 224.1 was obtained. The pulsed primary ion current was 0.29 pA and the primary ion dose density was 1.5 x $10^{12}$ ions/cm$^2$ in both ion modes. Large images were performed in the stage scan macroraster function with 2 shots per pixel and 4 frames over an area of 800 x 800 µm$^2$ to cover the entire fly head, and the resulting primary ion dose density was 7.7 x $10^{12}$ ions/cm$^2$.

**Data analysis**

The ToF-SIMS spectra and images were recorded and analyzed using the SURFACELAB 6 software (version 6.6, ION-TOF GmbH). The mass spectra were calibrated according to the peaks $[CH]^+$, $[CH_2]^+$, $[CH_3]^+$, $[C_2H_5N]^+$, and $[C_5H_{15}PNO_4]^+$ for positive ion mode, and $[CH_2]^-$, $[CH_3]^-$, $[C_2]^-$, $[C_{16}H_{31}O_2]^-$, $[C_{18}H_{33}O_2]^-$ for negative ion mode. The spectra were collected from the region of central fly brains and normalized to the number of pixels and the primary ion dose density.

Principal component analysis (PCA) was performed on ToF-SIMS spectra using SIMCA (Umetrix, Sweden). The region of interest (ROI) – the central fly brain – was chosen from each ToF-SIMS image. The spectra of ROI were normalized to the total number of selected pixels and the total ion intensity. This data set was applied pareto scaling for PCA analysis.

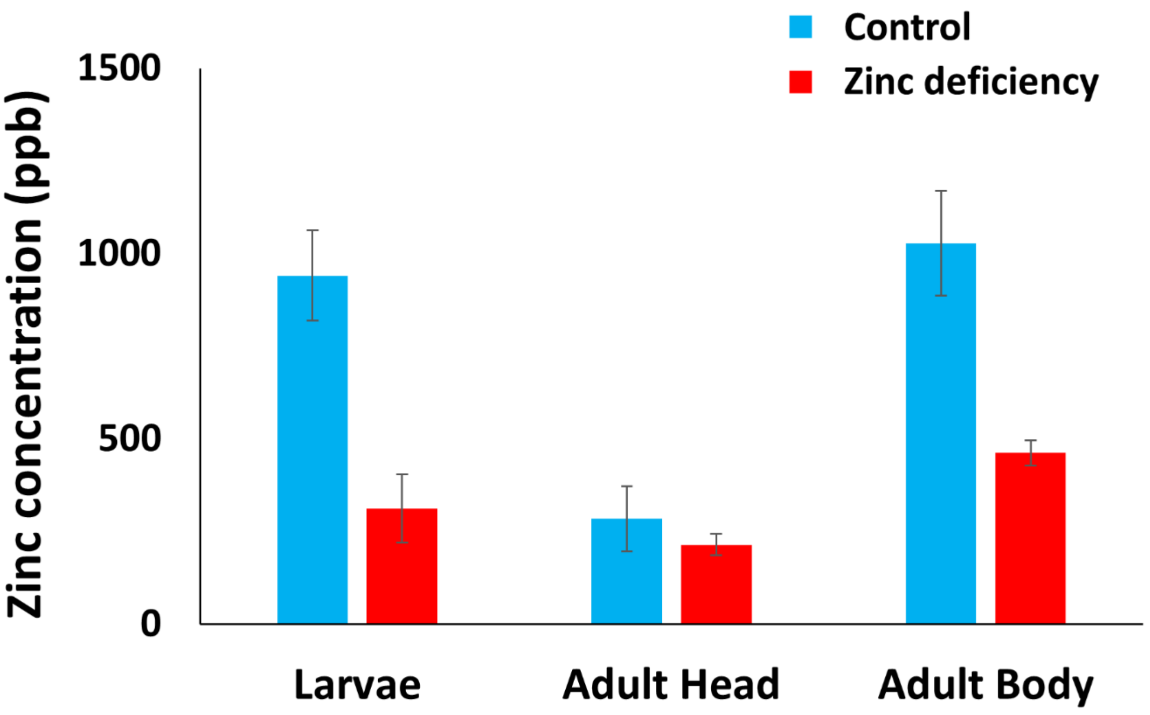


**Figure S1.** Average concentration of zinc in fly larvae, adult head and adult body analyzed by ICP-MS. Numbers of male adult flies analyzed for control and zinc deficient groups (adult body and head) were 28 and 22, respectively. Numbers of larvae analyzed by ICP-MS for control and zinc deficient groups were 23 and 30, respectively. The error bars represent SD.

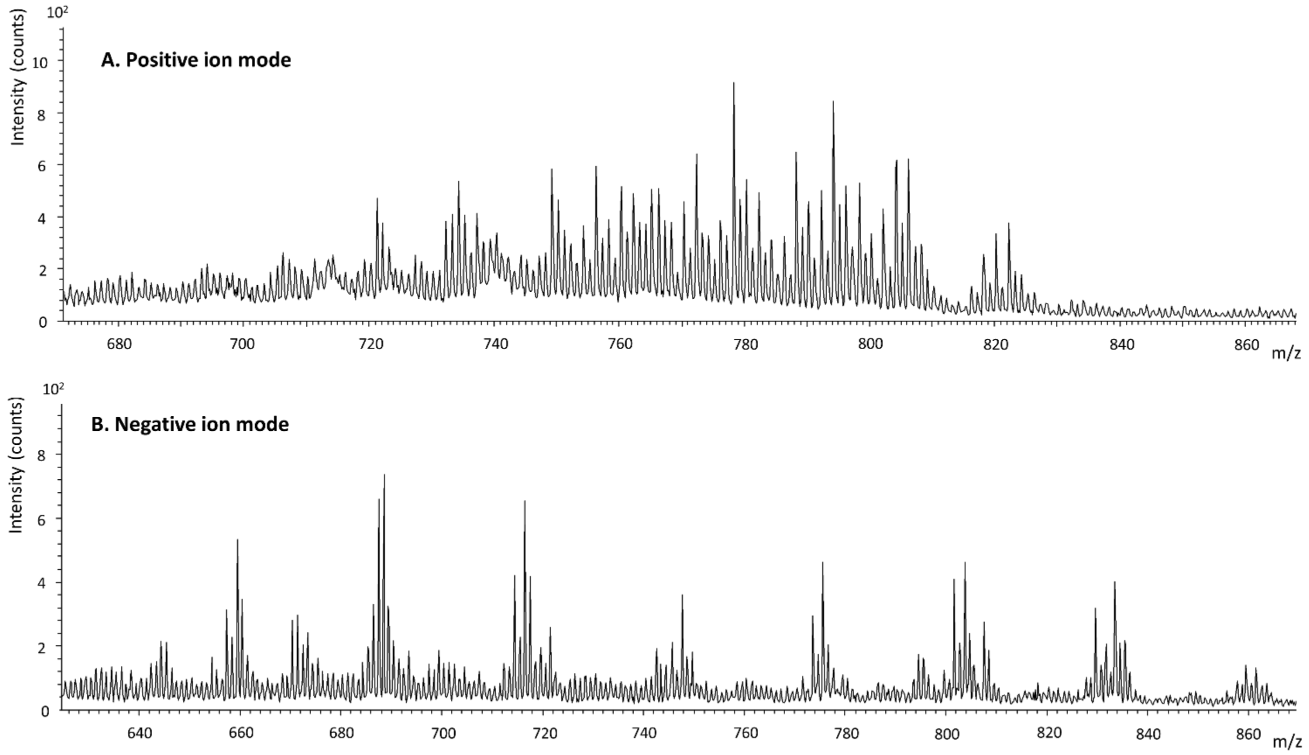


**Figure S2.** ToF-SIMS spectra of lipid regions in positive and negative ion modes.
